# Consistent Reduction in Periprocedural Myocardial Infarction With Cangrelor as Assessed by Multiple Definitions: Findings From CHAMPION PHOENIX (Cangrelor Versus Standard Therapy to Achieve Optimal Management of Platelet Inhibition)

**DOI:** 10.1161/CIRCULATIONAHA.115.020829

**Published:** 2016-09-05

**Authors:** Matthew A. Cavender, Deepak L. Bhatt, Gregg W. Stone, Harvey D. White, Ph. Gabriel Steg, C. Michael Gibson, Christian W. Hamm, Matthew J. Price, Sergio Leonardi, Jayne Prats, Efthymios N. Deliargyris, Kenneth W. Mahaffey, Robert A. Harrington

**Affiliations:** From University of North Carolina, Chapel Hill (M.A.C.); Brigham and Women’s Hospital, Heart and Vascular Center, Harvard Medical School, Boston, MA (D.L.B.); Columbia University, New York, NY (G.W.S.); University of Auckland, Auckland City Hospital, Auckland, New Zealand (H.D.W.); Université Paris-Diderot, Sorbonne Paris Cité, INSERM U-1148, DHU FIRE, Hópital Bichat, Assistance Publique-Hópitaux de Paris, Paris, France (P.G.S.); Institute of Cardiovascular Medicine and Science, National Lung and Heart Institute, Imperial College, Royal Brompton Hospital, London, United Kingdom (P.G.S.); Beth Israel Deaconess Medical Center, Harvard Medical School, Boston, MA (C.M.G.); Kerckhoff Clinic and Thoraxcenter, Bad Nauheim, Germany (C.W.H.); Scripps Clinic, La Jolla, CA (M.J.P.); UOC Fondazione IRCCS Policlinico San Matteo, Pavia, Italy (S.L.); The Medicines Company, Parsippany, NJ (J.P., E.N.D.); and Stanford University, Palo Alto, CA (K.W.M., R.A.H.).

**Keywords:** creatine kinase, MB form, myocardial infarction, percutaneous coronary intervention

## Abstract

**BACKGROUND:**

Cangrelor is an intravenous P2Y_12_ inhibitor approved to reduce periprocedural ischemic events in patients undergoing percutaneous coronary intervention not pretreated with a P2Y_12_ inhibitor.

**METHODS:**

A total of 11 145 patients were randomized to cangrelor or clopidogrel in the CHAMPION PHOENIX trial (Cangrelor versus Standard Therapy to Achieve Optimal Management of Platelet Inhibition). We explored the effects of cangrelor on myocardial infarction (MI) using different definitions and performed sensitivity analyses on the primary end point of the trial.

**RESULTS:**

A total of 462 patients (4.2%) undergoing percutaneous coronary intervention had an MI as defined by the second universal definition. The majority of these MIs (n=433, 93.7%) were type 4a. Treatment with cangrelor reduced the incidence of MI at 48 hours (3.8% versus 4.7%; odds ratio [OR], 0.80; 95% confidence interval [CI], 0.67–0.97; *P*=0.02). When the Society of Coronary Angiography and Intervention definition of periprocedural MI was applied to potential ischemic events, there were fewer total MIs (n=134); however, the effects of cangrelor on MI remained significant (OR, 0.65; 95% CI, 0.46–0.92; *P*=0.01). Similar effects were seen in the evaluation of the effects of cangrelor on MIs with peak creatinine kinase-MB ≥10 times the upper limit of normal (OR, 0.64; 95% CI, 0.45–0.91) and those with peak creatinine kinase-MB ≥10 times the upper limit of normal, ischemic symptoms, or ECG changes (OR, 0.63; 95% CI, 0.48–0.84). MIs defined by any of these definitions were associated with increased risk of death at 30 days. Treatment with cangrelor reduced the composite end point of death, MI (Society of Coronary Angiography and Intervention definition), ischemia-driven revascularization, or Academic Research Consortium definite stent thrombosis (1.4% versus 2.1%; OR, 0.69; 95% CI, 0.51–0.92).

**CONCLUSIONS:**

MI in patients undergoing percutaneous coronary intervention, regardless of definition, remains associated with increased risk of death in the current era. Cangrelor compared with clopidogrel significantly reduces MI regardless of the definition.

**CLINICAL TRIAL REGISTRATION:**

URL: http://clinicaltrials.gov. Unique identifier: NCT01156571.

## Abstract 2

Supplemental Digital Content is available in the text.

Cangrelor is a reversible, intravenous adenosine diphosphate receptor antagonist and has a rapid onset and offset of its antiplatelet effects. Cangrelor has been approved for use in patients undergoing percutaneous coronary intervention (PCI) not pretreated with a P2Y_12_ inhibitor on the basis of the results of the CHAMPION PHOENIX trial (Cangrelor versus Standard Therapy to Achieve Optimal Management of Platelet Inhibition).^1^ The CHAMPION PHOENIX trial compared cangrelor with clopidogrel in patients undergoing PCI and found that cangrelor reduced the composite end point of death, myocardial infarction (MI), ischemia-driven revascularization, or stent thrombosis by 22%.^1^ The definition of MI used in the trial was based on the second universal definition of MI, and the protocol mandated the collection of blood samples to test for biomarkers of myonecrosis after the initial PCI.^2^ A core laboratory also reviewed all angiograms from the index PCI to determine whether there was evidence of angiographic complications during the procedure, including intraprocedural stent thrombosis (IPST).^3^

Some have questioned whether MIs and IPST detected through these standardized efforts represent clinically meaningful events. In this analysis, we sought to determine whether changes in the definition of MI or stent thrombosis would have affected the overall primary end point of the trial. Additionally, we aimed to evaluate the effects of cangrelor on MIs of different sizes and types. Finally, we sought to better understand the impact of the different definitions on the incidence of MI and the prognosis of patients who have an MI.

## METHODS

### Study Population and Design

CHAMPION PHOENIX was a double-blind, placebo-controlled trial that randomized 11 145 patients who were undergoing PCI to either intravenous cangrelor or clopidogrel. The full inclusion and exclusion criteria and protocol of the trial have been reported previously.^1,4^ In brief, patients were eligible for the trial if they had not been treated previously with platelet inhibitors and were undergoing PCI for ST-segment–elevation MI, non–ST-segment–elevation acute coronary syndrome, or stable angina.

Patients were randomized to receive either cangrelor followed by clopidogrel after the infusion of cangrelor was complete or clopidogrel as soon as possible after randomization. Patients randomized to cangrelor received an infusion of cangrelor (30 μg/kg followed by an infusion of 4 μg/kg per minute) and placebo capsules. Cangrelor was continued for at least 2 hours or for the duration of the procedure (if the procedure lasted >2 hours) and was followed by clopidogrel 600 mg after the infusion of the intravenous study drug. Patients randomized to clopidogrel received either 600 or 300 mg clopidogrel as determined by clinician preference. Randomization was stratified by intended loading dose of clopidogrel (600 versus 300 mg) and normal or abnormal status at baseline (status based on cardiac biomarkers, changes in the ECG, and symptoms). Biomarkers of myonecrosis (creatinine kinase-MB fraction [CK-MB]; Siemens ADVIA Centaur 2-site sandwich immunoassay) were to be measured every 6 hours and analyzed by a core laboratory (Quest Diagnostics).

### End Points

A Clinical Events Committee that was independent, was unaware of the treatment assignments, and was based at the Duke Clinical Research Institute adjudicated all components of the primary and secondary efficacy end points. The primary efficacy end point was a composite of death, MI, ischemia-driven revascularization, or stent thrombosis at 48 hours. The definition of MI used in the adjudication of the primary efficacy end point was based on the second Universal Definition of MI and was the most contemporary definition available at the time in which the trial was designed (Table 1).^2^

**Table 1.**
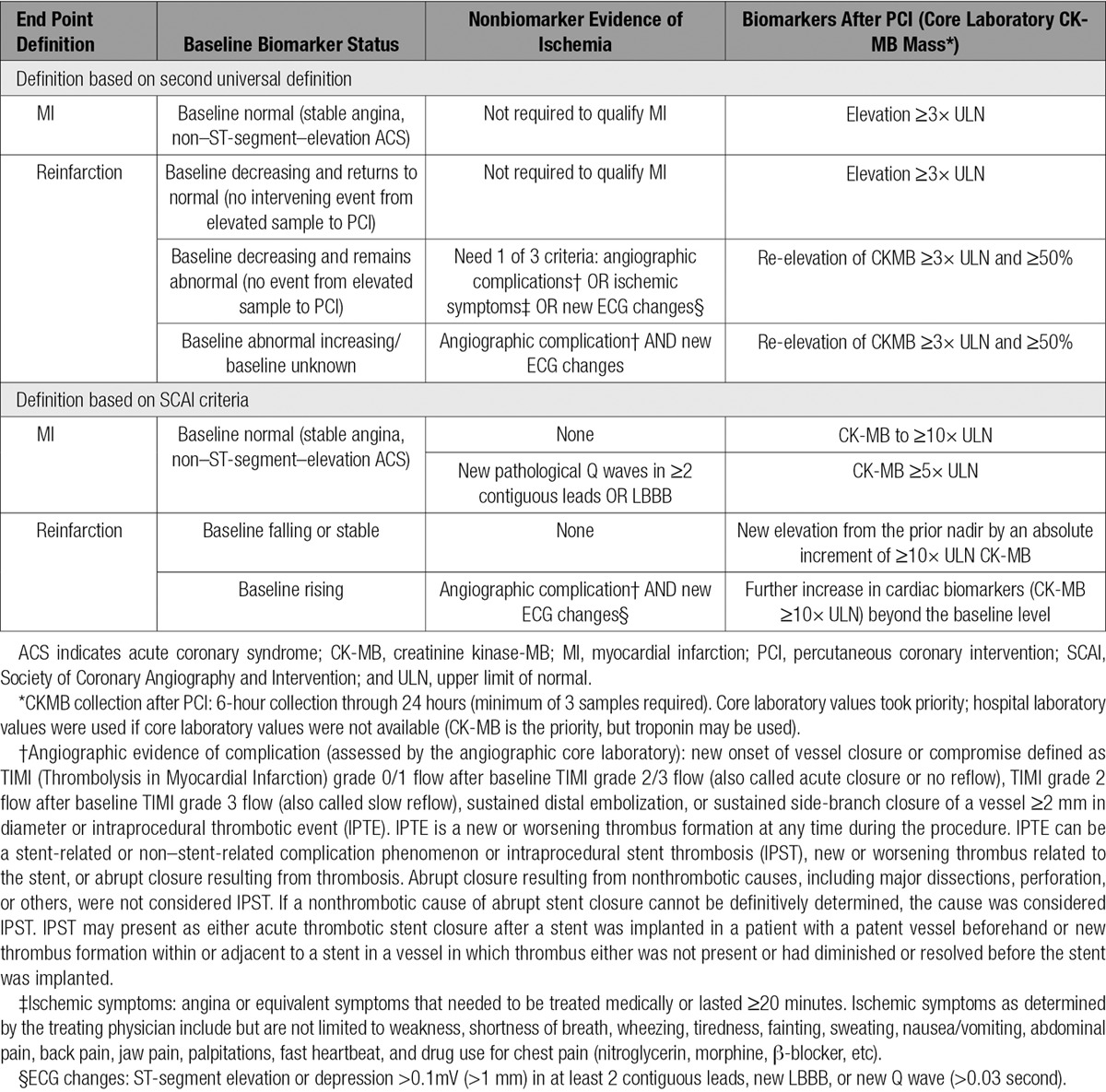
Definitions of PCI-Related (Type 4a) MI

To determine whether patients had a periprocedural MI within 48 hours of randomization, the Clinical Events Committee classified patients on the basis of their cardiac biomarker status at baseline. Patients were considered to be normal at baseline if they were undergoing PCI for stable angina, had baseline levels of cardiac biomarkers below the 99th percentile, and had no new ECG changes or symptoms consistent with acute coronary syndrome within the prior 6 hours. Additionally, patients were considered to be normal at baseline if they were classified as undergoing PCI for a non–ST-segment–elevation acute coronary syndrome but had 2 biomarker samples ≥6 hours apart that were below the 99th percentile, no new ECG changes, and no ongoing acute coronary syndrome symptoms or symptoms within 6 hours before the sample. In these patients who had no evidence of MI at baseline, MI was considered to have occurred if there was an elevation in CK-MB that was ≥3 times the upper limit of normal (ULN).

All other patients in the trial were considered to be having an MI at the time of PCI and were further categorized on the basis of whether biomarkers were decreasing and returned to normal, were decreasing but remaining abnormal, were increasing, or had an unknown baseline. Those patients in whom baseline biomarkers were decreasing and were beneath the ULN were considered to have a reinfarction if there was an elevation in CK-MB that was ≥3 times the ULN. Patients with biomarkers that were decreasing at baseline but remained above the ULN were considered to have a reinfarction if they had an angiographic complication, ischemic symptoms, or new ECG changes (new ST-segment elevation/depression >0.1mV in at least 2 contiguous leads; new left bundle-branch block; new Q wave [> 0.03 seconds]) in conjunction with a re-elevation of CK-MB that was ≥3 times the ULN and ≥ 50% higher than the nadir. Patients in whom biomarkers were increasing at baseline (including patients with only 1 elevated biomarker sample at baseline or 2 biomarkers values collected <6 hours apart that prevented the adequate assessment of the biomarker trajectory) had to have both angiographic evidence consistent with a periprocedural event (sustained acute vessel closure, new Thrombolysis in Myocardial Infarction grade 0/1 flow, evidence of distal embolization, side-branch closure off a vessel ≥2 mm in diameter, dissection, thrombus, or no reflow) and ischemic ECG changes in addition to a re-elevation of CK-MB that was ≥3 times the ULN and ≥50% from baseline. Patients determined to have a ST-segment–elevation MI at baseline (including those patients with normal baseline cardiac markers who are confirmed by the Clinical Events Committee to have a baseline ST-segment–elevation MI ECG) were not reviewed for potential type 4a MI.

The Society of Coronary Angiography and Intervention (SCAI) subsequently proposed a new definition for periprocedural MI.^5^ In this analysis, we retrospectively applied the SCAI criteria to those events adjudicated as an MI during the trial. In patients with normal cardiac biomarkers at baseline, patients had to have an elevation of CK-MB to ≥10 times the ULN. Patients who had normal biomarkers at baseline but developed new pathological Q waves in ≥2 contiguous leads or left bundle-branch block were considered to have an MI if they have a CK-MB elevation ≥5 times the ULN. Patients with cardiac biomarkers that were either stable or falling at baseline required a new elevation from the previous nadir level by an absolute increment of ≥10 times the ULN of CK-MB. Patients with biomarkers that were rising at baseline required a further increase in cardiac biomarkers (CK-MB ≥10 times the ULN) beyond the last level. In the population of patients with rising biomarkers, the SCAI definition also requires signs consistent with a clinically relevant MI (ie, new onset or worsening heart failure or sustained hypotension). These specific events were not prospectively collected in the trial. Instead, for this analysis, we required patients with biomarkers that were rising at baseline to have either new ischemic ECG changes or angiographic evidence consistent with a clinically relevant MI.

The predefined definition of stent thrombosis used in the original analysis of CHAMPION PHOENIX included both definite stent thrombosis (as defined by the Academic Research Consortium [ARC]) and IPST (angiographically confirmed new or worsening thrombus related to the placement of the coronary stent).^6,7^ The first sensitivity analysis excluded IPST and was the composite of death, MI, ischemia-driven revascularization, or ARC definite stent thrombosis at 48 hours after randomization. Additional sensitivity analysis also excluded IPST and used revised MI definitions based on SCAI’s proposed clinically relevant threshold for an MI (death, MI [SCAI definition for peri-procedural events], ischemia-driven revascularization, or ARC definite stent thrombosis) and a definition of MI that required a postprocedural CK-MB elevation of ≥10 times the ULN (death, MI [CK-MB ≥10 times the ULN], ischemia-driven revascularization, or ARC definite stent thrombosis) at 48 hours after randomization.

An academic executive committee and The Medicines Company designed the trial. The Medicines Company funded the trial. At the conclusion of the trial, the database was transferred to the Harvard Clinical Research Institute, which had full access to the data and validated the analyses included in this article. The Institutional Review Board or Ethics Committee for each participating institution reviewed and approved the trial. All patients provided written informed consent.

### Statistical Analysis

This analysis used the modified intention to treat population of patients who underwent PCI and were treated with study drug (n=10 942). The association between MI type and the primary efficacy end point was examined with a logistic regression model that controlled for potential confounders (treatment, patient status, age [≥65 years], history of congestive heart failure, diabetes mellitus, prior MI, country [United States/Non–United States], PCI duration).

The effects of cangrelor compared with clopidogrel on MI were determined with a logistic regression model that was stratified by intended loading dose of clopidogrel (600 versus 300 mg) and patient status at baseline (normal/abnormal).

A 2-sided significance level of 0.05 was used, and there were no corrections for multiple comparisons because of the exploratory nature of this analysis. Event curves were developed from 48-hour Kaplan-Meier estimates. Analyses were performed with SAS software version 9.3 (SAS Institute, Cary, NC).

## RESULTS

**Figure 1.**
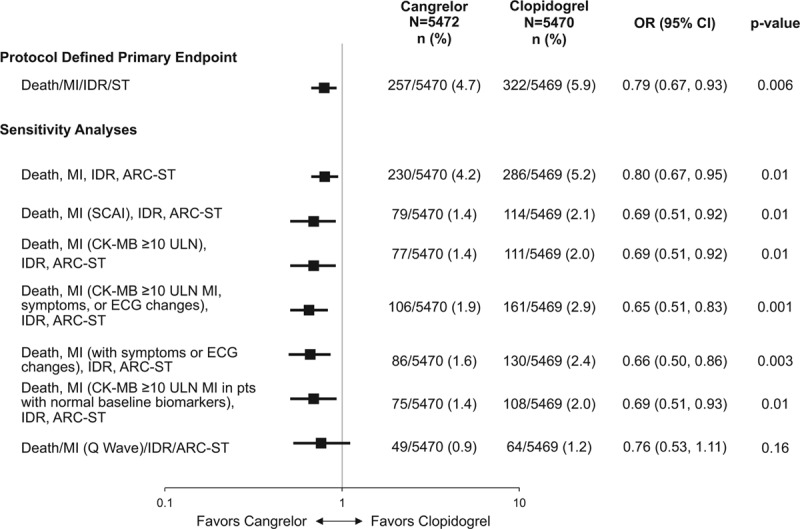
**Protocol-defined and sensitivity analyses of primary efficacy end points at 48 hours using different definitions of myocardial infarction (MI).**ARC indicates Academic Research Consortium; CI, confidence interval; CK-MB, creatinine kinase-MB; IDR, ischemia-driven revascularization; OR, odds ratio; SCAI, Society of Coronary Angiography and Intervention; ST, stent thrombosis; and ULN, upper limit of normal.

**Figure 2.**
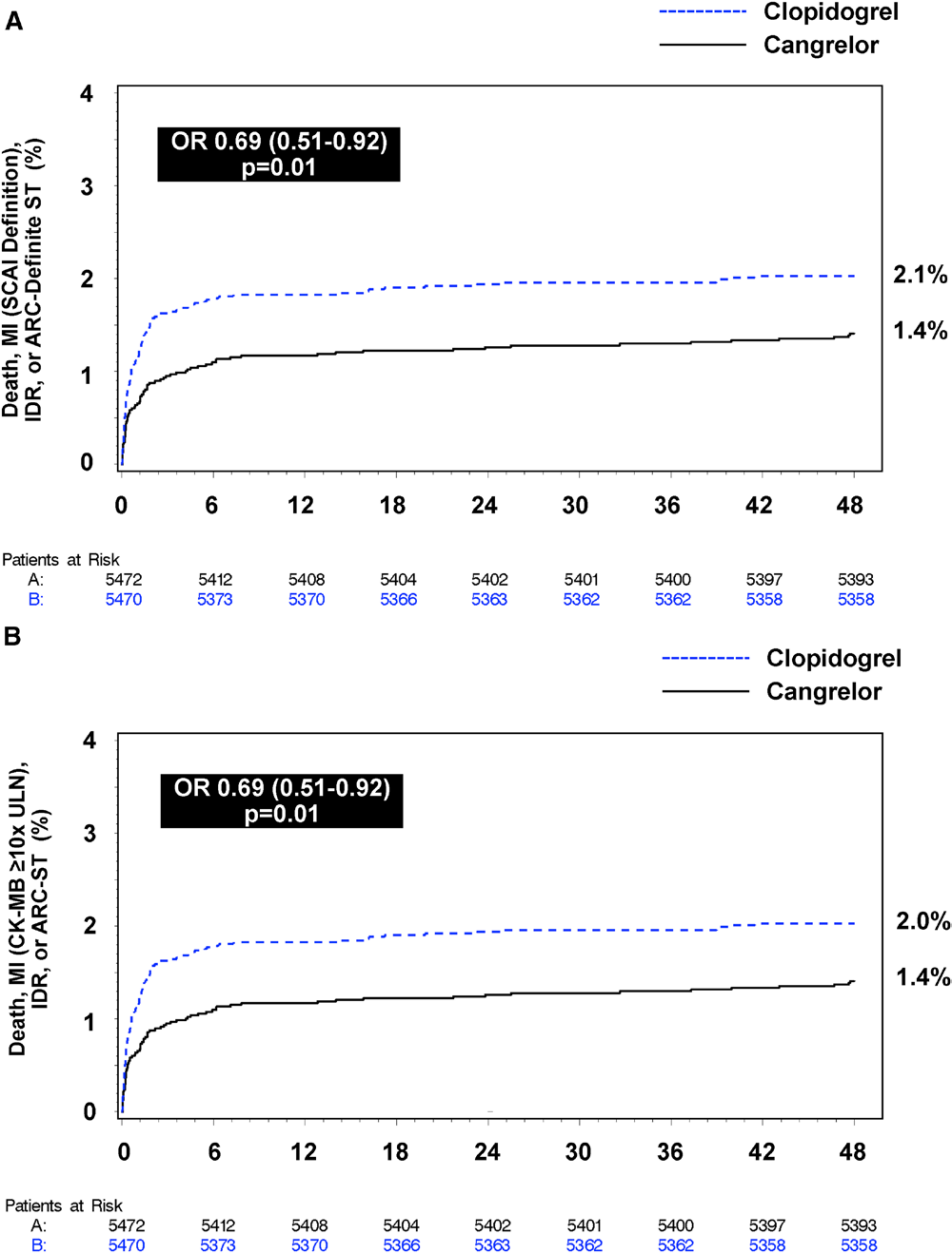
**Sensitivity analyses evaluating outcomes at 48 hours of cangrelor compared with clopidogrel using different definitions of myocardial infarction (MI).**Time to first occurrence of (**A**) death, MI (Society of Coronary Angiography and Intervention [SCAI] definition), ischemia-driven revascularization (IDR), and Academic Research Consortium (ARC) definite stent thrombosis (ST) and (**B**) death, MI (creatinine kinase-MG [CK-MB] ≥10 times the upper limit of normal [ULN]), IDR, and ARC definite stent thrombosis. OR indicates odds ratio.

The primary efficacy end point as originally defined in the CHAMPION PHOENIX trial was the composite of death, MI, ischemia-driven revascularization, or stent thrombosis at 48 hours. In this sensitivity analysis in which IPST was not included as part of the stent thrombosis events considered in the composite end point, cangrelor reduced death, MI, ischemia-driven revascularization, or ARC definite stent thrombosis at 48 hours (4.2% versus 5.2%; odds ratio [OR], 0.80; 95% confidence interval [CI], 0.67–0.95; *P*=0.01; Figure 1). When different definitions of MI were used in the primary efficacy end point, the results of the trial were consistent with previously reported results. Treatment with cangrelor reduced the composite end point of death, MI (using SCAI definition for periprocedural MI), ischemia-driven revascularization, or ARC definite stent thrombosis (1.4% versus 2.1%; OR, 0.69; 95% CI, 0.51–0.92; *P*=0.01) and the composite end point of death, MI (peak CK-MB ≥10 times the ULN), ischemia-driven revascularization, or ARC definite stent thrombosis (1.4% versus 2.0%; OR, 0.69; 95% CI, 0.51–0.92; *P*=0.01; Figure 2).

Treatment with cangrelor reduced the incidence of MI at 48 hours (3.8% versus 4.7%; OR, 0.80; 95% confidence interval [CI], 0.67–0.97; *P*=0.02). Of the 462 patients with MI that occurred in CHAMPION PHOENIX (4.2%), the majority were considered type 4a (related to PCI; n=433, 93.7%) and occurred in patients with baseline biomarkers that were normal (Table I in the online-only Data Supplement). Treatment with cangrelor reduced the incidence of type 4a MI compared with clopidogrel (3.5% versus 4.4%; OR, 0.80; 95% CI, 0.66–0.98; *P*=0.03; Table 2). When the SCAI definition of periprocedural MI was used for potential ischemic events that occurred during the trial, there were fewer overall events (n=134). Treatment with cangrelor also reduced the incidence of MI using the SCAI criteria for periprocedural events (1.0% versus 1.5%; OR, 0.65; 95% CI, 0.46–0.92; *P*=0.01). In patients with baseline biomarkers that were normal, cangrelor reduced the incidence of MIs with symptoms and CK-MB ≥5 times the ULN (0.4% versus 0.7%; OR, 0.54; 95% CI, 0.32–0.91; *P*=0.02). Similarly, cangrelor reduced MIs with a peak CK-MB ≥10 times the ULN (0.9% versus 1.4%; OR, 0.64; 95% CI, 0.45–0.91; *P*=0.01) and those MI with peak CK-MB ≥10 times the ULN, symptoms, or ECG changes (1.5% versus 2.4%; OR, 0.63; 95% CI, 0.48–0.84; *P*=0.001). There was no heterogeneity in the effects of cangrelor on MI based on index diagnosis (Table II in the online-only Data Supplement). The distribution of peak biomarkers in patients treated with cangrelor and clopidogrel is shown in Figure I in the online-only Data Supplement.

**Table 2.**
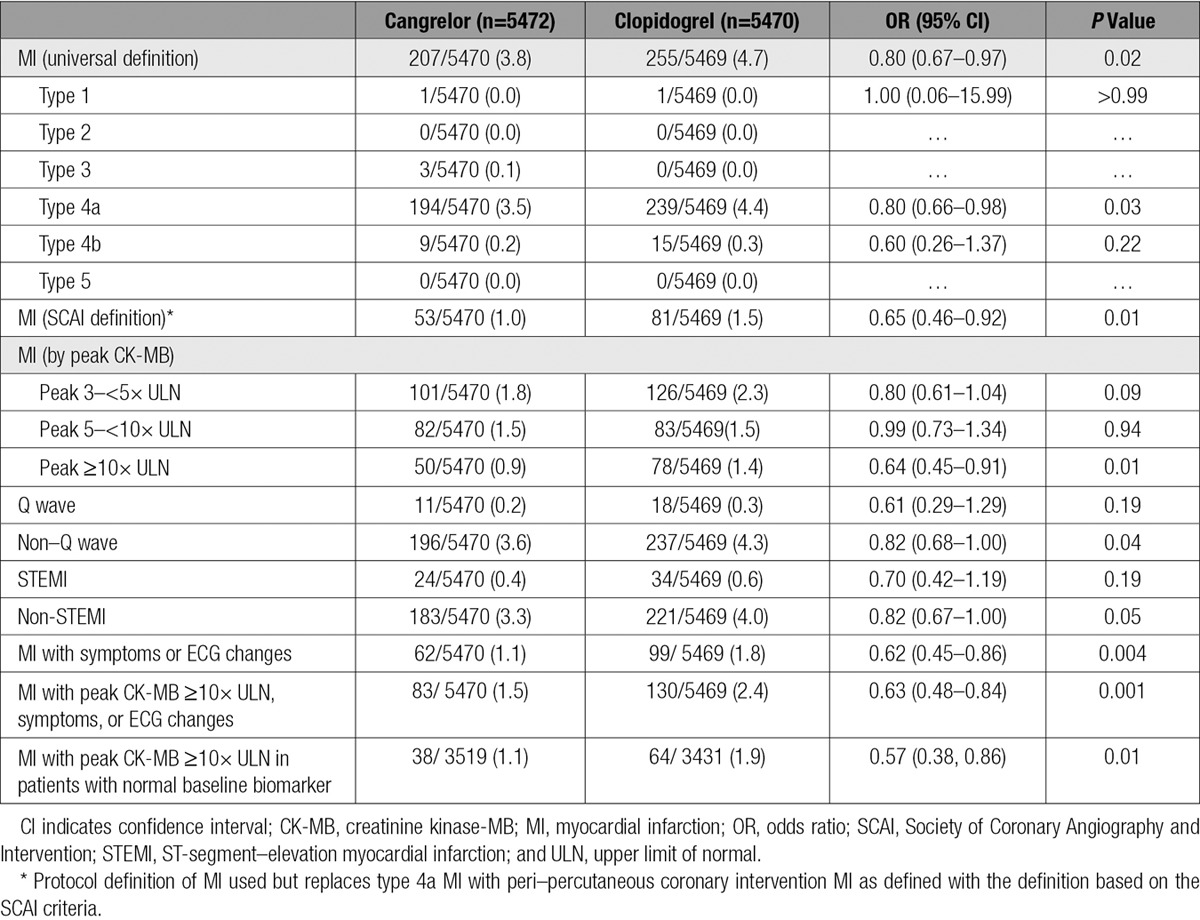
Effect of Cangrelor on MI at 48 Hours

**Figure 3.**
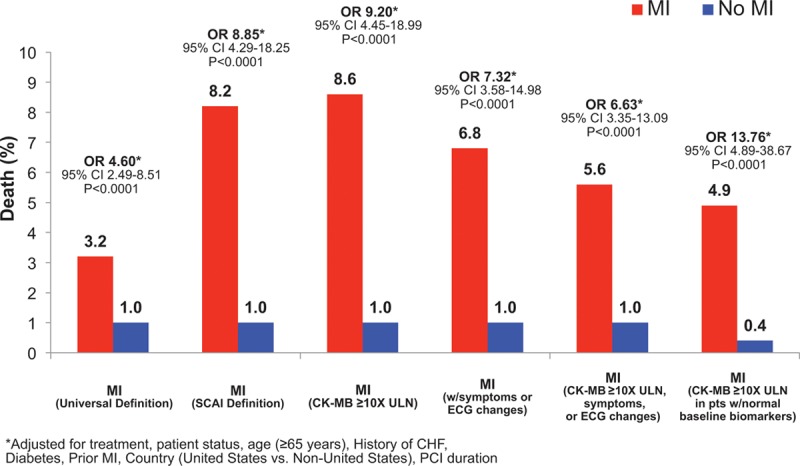
**Association between the types of myocardial infarction (MI) using various definitions and the risk of death at 30 days.**CHF indicates coronary heart failure; CI, confidence interval; CK-MB, creatinine kinase-MB; MI, myocardial infarction; OR, odds ratio; PCI, percutaneous coronary intervention; SCAI, Society of Coronary Angiography and Intervention; and ULN, upper limit of normal.

Patients randomized in CHAMPION PHOENIX who had an MI as defined with the definition in the original protocol that was based on the second universal definition of MI were at increased risk of death at 30 days even after adjustment for potential confounders (adjusted OR, 4.60; 95% CI, 2.49–8.51; *P*<0.001; Figure 3). MI also was associated with increased risk of death at 30 days when using the SCAI definition (adjusted OR, 8.85; 95% CI, 4.29–18.25; *P*<0.001) and when considering only MI that resulted in a peak CK-MB ≥10 times the ULN (adjusted OR, 9.20; 95% CI, 4.45–18.99; *P*<0.001).

## DISCUSSION

In this sensitivity analysis of the CHAMPION PHOENIX trial, we found that treatment with cangrelor reduced the incidence of death, MI, ischemia-driven revascularization, or stent thrombosis regardless of the definition of MI or stent thrombosis. The consistency of the results across a variety of different definitions supports the efficacy of cangrelor in patients undergoing PCI. Furthermore, cangrelor reduced MIs of different sizes, but particularly those MIs associated with high levels of biomarkers and MIs that were associated with signs or symptoms of ischemia. We also found that the occurrence of an MI, regardless of type, remains associated with significant odds of death in the 30 days after the event. Thus, the benefits of cangrelor in reducing periprocedural MI remain clinically important in the current era.

Because the majority of ischemic events that occurred during the first 48 hours of CHAMPION PHOENIX were type 4a MIs related to PCI, these data highlight the need for adequate and effective antithrombotic therapies.^8^ Current guidelines recommend that patients undergoing PCI be treated with oral antiplatelet therapies before or immediately after the procedure.^9–11^ The oral P2Y_12_ inhibitors currently used in practice have a delayed onset of action and do not result in antiplatelet effects for ≈60 to 120 minutes.^12,13^ The delay in the onset of antiplatelet effect is a particular problem in patients such as those treated with ad hoc PCI after elective coronary angiography or in patients undergoing PCI for acute coronary syndrome in whom little antiplatelet effect may be present during the actual PCI. Furthermore, some medications such as those used during moderate sedation may delay the onset of antiplatelet effects.^14^

Intravenous glycoprotein IIb/IIIa inhibitors are available and can be used in patients undergoing PCI who are not pretreated with an oral P2Y_12_ inhibitor.^15^ However, prior studies showed that the majority of benefit from glycoprotein IIb/IIIa inhibitors occurred in patients with elevated cardiac biomarkers, yet the EARLY-ACS trial (Early Glycoprotein IIb/IIIa Inhibition in Non–ST-Segment Elevation Acute Coronary Syndrome) and ACUITY trial (Acute Catheterization and Urgent Intervention Triage Strategy) showed that the routine use of these agents in these populations increased the risk of bleeding without significant improvements in clinical outcomes.^16,17^ As a result, the use of glycoprotein IIb/IIIa inhibitors has declined, and some current guidelines do not endorse routine use.^9^ Because cangrelor is an intravenous formulation that has high bioavailability and is a highly effective platelet inhibitor, it is likely that much of the benefit with regard to the reduction of MI seen in this study reflects an antiplatelet effect that is more potent and occurs more quickly than the effects of clopidogrel.^18^ Prior studies have shown that more intensive antiplatelet therapy can reduce ischemic events in patients undergoing PCI.^10,11,19^ These findings from CHAMPION PHOENIX build on prior studies and show that drugs with greater bioavailability and a faster onset of action can reduce ischemic events even compared with effective antiplatelet therapies.^20–23^

Some have questioned whether MIs, particularly those events with low elevations in cardiac biomarkers that may not have been previously detected, are clinically relevant in the contemporary era.^24,25^ We found that patients with an MI after randomization had a risk of death that was between 4- and 13-fold greater than that for patients who had no event. The clear association between MI and death, which was present for all definitions of MI evaluated in these analyses, provides evidence that therapies designed to reduce MIs are likely important in the treatment of patients undergoing PCI.

Numerous definitions of MI have been proposed for use in clinical trials^2,5,26^; however, the optimal definition remains unclear.^7,27^ Furthermore, differences in the definitions change the incidence of periprocedural MI as noted by the fact that only 31% of the type 4a MIs as defined by the second universal definition met the SCAI criteria. The second universal definition of MI noted that it was difficult to define MI in patients with elevated biomarkers at baseline. These guidelines suggested including criteria that incorporate features supportive of ischemia (eg, imaging, ECG). In CHAMPION PHOENIX, patients with elevated biomarkers required evidence of symptoms, angiographic evidence consistent with a periprocedural event, or ischemic ECG changes, in addition to further elevations in biomarkers, to be considered as having a type 4a MI. In patients who had no evidence of MI at baseline, MI was considered to have occurred if there was an elevation in CK-MB that was ≥3 times the ULN regardless of whether symptoms were present. The third universal definition now requires that patients have signs or symptoms of myocardial ischemia regardless of baseline biomarkers. In addition, the third universal definition proposes a >5-fold rise above the 99th percentile for the upper reference limit for patients with normal biomarkers at baseline to meet the MI definition.^26^

The SCAI definition differs in some important ways from the universal definition. Patients with normal cardiac biomarkers at baseline had to have an elevation of CK-MB to ≥10 times the ULN or cardiac troponin (I or T) to ≥70 times the ULN to meet the definition of MI. Although this definition identified fewer events in CHAMPION PHOENIX, the events identified were clinically meaningful and were associated with increased risk of death at 30 days even when controlling for the extent of coronary artery disease.

These data should be considered in light of some limitations. First, these analyses are post hoc analyses that were not prespecified. As a result, they represent sensitivity analyses that supplement the findings from the predefined, primary results of the trial. Accordingly, the MI definition based on the SCAI criteria was not prospectively adjudicated and was obtained retrospectively. This trial assessed MI using the universal definition that was available at that time the trial was designed and did not use the third universal definition that is now available. In addition, CK-MB was systematically collected in the CHAMPION PHOENIX trial, whereas the second and third universal definitions of MI prefer cardiac troponin. Bleeding was investigator reported and was not adjudicated, although post hoc adjudication did not qualitatively change the safety profile.^23^ Finally, CHAMPION PHOENIX was not powered for this specific component of the primary end point or to assess the effects of cangrelor on specific types of MI.

## CONCLUSIONS

MI, as defined with a variety of different classifications, continues to be an important event in the contemporary era that significantly increases the risk of death at 30 days. Although there are multiple proposed definitions of MI and these different definitions result in changes in the incidence of MI, there were no qualitative differences in the effects of cangrelor with the use of various definitions. As a result, cangrelor was effective in reducing ischemic events in patients undergoing PCI in CHAMPION PHOENIX regardless of the definition of MI tested in this study.

## ACKNOWLEDGMENTS

We would like to thank Steven E. Elkin, MS, and Debra Bernstein, PhD, of The Medicines Company for their statistical support, along with Yuyin Liu, MS, and Lanyu Lei, MS, of the Harvard Clinical Research Institute for their independent verification of the analyses. Harvard Clinical Research Institute received funding from The Medicines Company for these analyses.

## SOURCE OF FUNDING

The CHAMPION PHOENIX trial was funded by The Medicines Company.

## DISCLOSURES

Dr Cavender reports consulting fees from AstraZeneca and Merck.

Dr Bhatt has served on the Advisory Board for Cardax, Elsevier Practice Update Cardiology, Medscape Cardiology, and Regado Biosciences; on the Board of Directors for Boston VA Research Institute and Society of Cardiovascular Patient Care; as chair for the American Heart Association Quality Oversight Committee; and on the Data Monitoring Committees for Duke Clinical Research Institute, Harvard Clinical Research Institute, Mayo Clinic, and Population Health Research Institute. Dr Bhatt has received honoraria from the American College of Cardiology (senior associate editor, *Clinical Trials and News*, ACC.org), Belvoir Publications (editor in chief, *Harvard Heart Letter*), Duke Clinical Research Institute (clinical trial steering committees), Harvard Clinical Research Institute (clinical trial steering committee), HMP Communications (editor in chief, *Journal of Invasive Cardiology*), *Journal of the American College of Cardiology* (guest editor; associate editor), Population Health Research Institute (clinical trial steering committee), Slack Publications (chief medical editor, *Cardiology Today’s Intervention*), Society of Cardiovascular Patient Care (secretary/treasurer), and WebMD (CME steering committees); other funding from *Clinical Cardiology* (deputy editor), NCDR-ACTION Registry Steering Committee (vice-chair), VA CART Research and Publications Committee (chair); research funding from Amarin, Amgen, AstraZeneca, Bristol-Myers Squibb, Eisai, Ethicon, Forest Laboratories, Ischemix, Medtronic, Pfizer, Roche, Sanofi Aventis, The Medicines Company (including for serving as co-chair of CHAMPION PHOENIX); and royalties from Elsevier (editor, *Cardiovascular Intervention: A Companion to Braunwald’s Heart Disease*). Dr Bhatt was a site coinvestigator for Biotronik, Boston Scientific, and St. Jude Medical; was a trustee for the American College of Cardiology; and performed unfunded research for FlowCo, PLx Pharma, Takeda.

Dr Stone reports no relevant disclosures.

Dr White reports honoraria from AstraZeneca and research funding from Sanofi-Aventis, Eli Lilly, National Health Institute, Glaxo Smith Kline, Merck Sharpe & Dohme, and AstraZeneca.

Dr Steg has received a research grant (to INSERM U1148) from Sanofi and Servier; has received speaking or consulting fees from Amarin, AstraZeneca, Bayer, Boehringer-Ingelheim, Bristol-Myers-Squibb, CSL-Behring, Daiichi-Sankyo, GlaxoSmithKline, Janssen, Lilly, Novartis, Pfizer, Regeneron, Roche, Sanofi, Servier, The Medicines Company; and owns stock in Aterovax.

Dr Gibson has received honoraria from The Medicines Company.

Dr Hamm has received honoraria from AstraZeneca, Sanofi Aventis, and Lilly, as well as research funding from Astra Zeneca and The Medicines Company.

Dr Price reports honoraria from AstraZeneca, Merck & Co, Accriva Diagnostics, and The Medicines Company.

Dr Leonardi has served on the Advisory Board for The Medicines Company, Eli Lilly, and Merck.

Drs Prats and Deliargyris are employed by The Medicines Company.

Dr Mahaffey has received honoraria from Bayer, Boehringer Ingelheim, Bristol Myers Squibb, Cubist, Eli Lilly, Epson, Forest, Glaxo Smith Kline, Johnson & Johnson, Medtronic, Merck, Mt. Sinai, Myokardia, Omthera, Portola, Purdue Pharma, Spring Publishing, Vindico, and WebMD, as well as research funding from Daiichi, Johnson & Johnson, Medtronic, St. Jude, and Tenax.

Dr Harrington has served on the Advisory Board for Evidint, Regado, and Scanadu; has received honoraria from Amgen, Daiichi-Lilly, Gilead Sciences, Gilead Sciences Inc, Janssen R&D, Medtronic, Merck, Novartis Corporation, The Medicines Company, Vida Health, Vox Media, and WebMD; has received other funding from the American Heart Association; has received research funding from AstraZeneca, BMS, CSL Behring, GSK, Merck, Portola, Sanofi-Aventis, and The Medicines Company; and has ownership interest in Element Science and MyoKardia.

## Supplementary Material

## Clinical Perspective

### What Is New?

- Cangrelor is an intravenous P2Y_12_ inhibitor approved to reduce periprocedural ischemic events in patients undergoing percutaneous coronary intervention not previously treated with a P2Y_12_ inhibitor.
- 4.2% of patients had a myocardial infarction (MI) as defined by the second universal definition within 48 hours after undergoing percutaneous coronary intervention.
- Cangrelor reduced MIs regardless of whether the MI was defined with the second universal or Society of Coronary Angiography and Intervention definition or when MIs were restricted to only those with large biomarker elevations and either symptoms or ECG changes.

### What Are the Clinical Implications?

- Changes in the definition of MI used in the primary end point did not affect the overall findings from the CHAMPION PHOENIX trial (Clinical Trial Comparing Cangrelor to Clopidogrel Standard of Care Therapy in Subjects Who Require Percutaneous Coronary Intervention).
- Some have questioned whether MI remains an important clinical event in the current era; however, we found that patients who had an MI, regardless of the definition, were at increased risk of death at 30 days.
- These findings demonstrate the importance of therapies that reduce ischemic events in patients undergoing percutaneous coronary intervention and provide further evidence for the clinical utility of cangrelor when used in patients undergoing percutaneous coronary intervention.
