# Supplementary material for: Consistent Reduction in Periprocedural Myocardial Infarction With Cangrelor as Assessed by Multiple Definitions: Findings From CHAMPION PHOENIX (Cangrelor Versus Standard Therapy to Achieve Optimal Management of Platelet Inhibition)
Source: Circulation. 2016 Sep 5;134(10):723–33. doi: 10.1161/CIRCULATIONAHA.115.020829 (PMC5006794; doi:10.1161/CIRCULATIONAHA.115.020829)

Supplemental Material

Supplemental Table I – Type 4a myocardial infarctions in CHAMPION PHOENIX in patients categorized by baseline biomarker status.

Endpoint	Subgroup	Cangrelor (N=5472)	Clopidogrel (N=5470)	Total (N=10942)
Type 4a	Overall	194/ 5470 (3.5)	239/5469 (4.4)	433/10939 (4.0)
	Baseline biomarkers normal or decreasing but returned to normal	181/194 (93.3)	217/239 (90.8)	398/433 (91.9)
	Biomarkers abnormal and increasing	6/194 (3.1)	11/239 (4.6)	17/433 (3.9)
	Baseline biomarkers abnormal and decreasing but remain abnormal	7/194 (3.6)	11/239 (4.6)	18/433 (4.2)
Type 4a (SCAI)	Overall	40/5470 (0.7)	66/5469 (1.2)	106/10939 (1.0)
	Baseline biomarkers normal or decreasing but returned to normal	33/40 (82.5)	56/65 (86.2)	89/105 (84.8)
	Biomarkers abnormal and increasing	2/40 (5.0)	6/65 (9.2)	8/105 (7.6)
	Baseline biomarkers abnormal and decreasing but remain abnormal	5/40 (12.5)	4/65 (6.2)	9/105 (8.6)
Type 4a MI with >10x elevation in biomarkers	Overall	37/5470 (0.7)	63/5469 (1.2)	100/10939 (0.9)
	Baseline biomarkers normal or decreasing but returned to normal	31/37 (83.8)	53/62 (85.5)	84/99 (84.8)
	Biomarkers abnormal and increasing	2/37 (5.4)	6/62 (9.7)	8/99 (8.1)
	Baseline biomarkers abnormal and decreasing but remain abnormal	4/37 (10.8)	4/62 (6.5)	8/99 (8.1)
Type 4a MI with >10x elevation in biomarkers plus symptoms and/or ECG change	Overall	11/16 (68.8)	23/31 (74.2)	34/47 (72.3)
	Baseline biomarkers normal or decreasing but returned to normal	2/16 (12.5)	5/31 (16.1)	7/47 (14.9)
	Biomarkers abnormal and increasing	3/16 (18.8)	4/31 (12.9)	7/47 (14.9)
	Baseline biomarkers abnormal and decreasing but remain abnormal	9/5470 (0.2)	16/5469 (0.3)	25/10939 (0.2)
Type 4a (Q Wave MI)	Overall	8/ 5470 (0.1)	12/ 5469 (0.2)	20/10939 (0.2)
	Baseline biomarkers normal or decreasing but returned to normal	3/8 (37.5)	9/12 (75.0)	12/20 (60.0)
	Biomarkers abnormal and increasing	2/8 (25.0)	3/12 (25.0)	5/20 (25.0)
	Baseline biomarkers abnormal and decreasing but remain abnormal	3/8 (37.5)	0/12 (0.0)	3/20 (15.0)

Supplemental Table II – Effects of cangrelor on MI based on index diagnosis.

	**Cangrelor n/N (%)**	**Clopidogrel n/N (%)**	**OR (95% CI)**	**p-value**	**p-value (interaction)**
**MI**					***0.32***
Stable Angina	169/3120 (5.4)	207/3018 (6.9)	0.78 (0.63-0.96)	0.02	
NSTE-ACS	33/1389 (2.4)	35/1421 (2.5)	0.96 (0.60-1.56)	0.88	
STEMI	5/961 (0.5)	13/1030 (1.3)	0.41 (0.15-1.15)	0.08	
**Type 4a**					***0.63***
Stable Angina	163/3120 (5.2)	199/3018 (6.6)	0.78 (0.63-0.97)	0.02	
NSTE-ACS	27/1389 (1.9)	31/1421 (2.2)	0.89 (0.53-1.50)	0.66	
STEMI	4/961 (0.4)	9/1030 (0.9)	0.47 (0.15-1.54)	0.21	
**SCAI**					***0.39***
Stable Angina	35/3120 (1.1)	57/3018 (1.9)	0.59 (0.39-0.90)	0.01	
NSTE-ACS	15/1389 (1.1)	16/1421 (1.1)	0.96 (0.47-1.95)	0.91	
STEMI	3/961 (0.3)	8/1030 (0.8)	0.40 (0.11-1.51)	0.16	

Presentation (stable angina, NSTE-ACS, STEMI) based on final designations.

Supplemental Figure I – Distribution of peak CK-MB in patients who had a Type 4a myocardial infarction treated with A) cangrelor and B) clopidogrel.

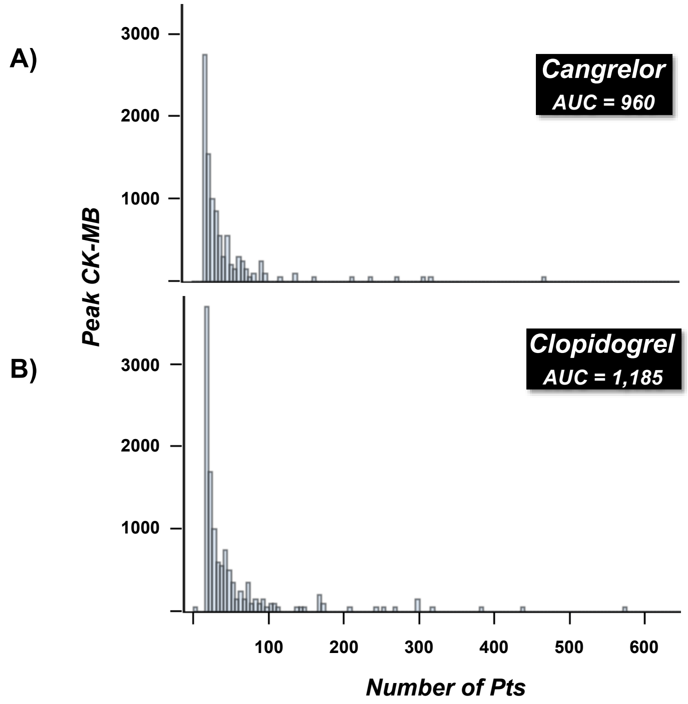

Supplement: Supplementary file 1 [file cir-134-723-s001.pdf]
